# Serum Aquaporin 4-Immunoglobulin G Titer and Neuromyelitis Optica Spectrum Disorder Activity and Severity: A Systematic Review and Meta-Analysis

**DOI:** 10.3389/fneur.2021.746959

**Published:** 2021-10-20

**Authors:** Jia Liu, Guojun Tan, Bin Li, Jingze Zhang, Ying Gao, Yuanbo Cao, Zhen Jia, Kazuo Sugimoto

**Affiliations:** ^1^Department of Neurology, Dongzhimen Hospital, Beijing University of Chinese Medicine, Beijing, China; ^2^Institute for Brain Disorders, Beijing University of Chinese Medicine, Beijing, China; ^3^Department of Neurology, The Second Hospital of Hebei Medical University, Shijiazhuang, Hebei, China

**Keywords:** neuromyelitis optica spectrum disorder, aquaporin4-immunoglobulin G, biomarker, clinical relevance, meta-analysis

## Abstract

**Background:** Aquaporin 4-immunoglobulin G (AQP4-IgG) plays a major role in the pathogenesis of neuromyelitis optica spectrum disorder (NMOSD). Seropositive status for this antibody has become one of the required indicators for NMOSD diagnosis.

**Objective:** Our goal was to systematically review and perform a meta-analysis of the current works of literature evaluating the clinical relevance of serum AQP4-IgG titer in patients with NMOSD. We sought to determine whether AQP4-IgG could indicate disease activity or severity, in addition to its diagnostic value in NMOSD.

**Methods:** Electronic databases were searched for published literature, yielding 4,402 hits. Of the 124 full articles screened, 17 were included in the qualitative analysis and 14 in the meta-analysis.

**Results:** There were no significant differences in serum AQP4-IgG titers between the relapse and remission phases in patients with NMOSD [standard mean difference (SMD): 0.32, 95% CI (−0.10, 0.74), *p* = 0.14]. Subgroup meta-analysis of AQP4-IgG detected by cell-based assays (CBA), an AQP4-IgG testing method recommended by the 2015 international consensus diagnostic criteria for NMOSD, confirmed the aforementioned result [SMD: 0.27, 95% CI (−0.01, 0.55), *p* = 0.06]. Moreover, the serum AQP4-IgG titer was positively correlated with the number of involved spinal cord segments [correlation coefficient (COR): 0.70, 95% CI (0.28–0.89), *p* = 0.003] and the Expanded Disability Status Scale (EDSS) score [COR: 0.54, 95% CI (0.06-0.82), *p* = 0.03] in the attack phase in patients with NMOSD.

**Conclusions:** The present study systematically assessed the association between serum AQP4-IgG titer and NMOSD activity and severity. The results demonstrated that the serum AQP4-IgG titer was not associated with disease activity but indicated the disease severity in the attack phase in patients with NMOSD. A further meta-analysis with a larger number of studies that employed standardized AQP4-IgG assays and detected attack–remission paired samples from the same patients with detailed medication information will be required to confirm our findings and shed more light on optimizing clinical AQP4-IgG monitoring.

**Systematic Review Registration:** [www.crd.york.ac.uk/PROSPERO/display_record.php?RecordID=208209], PROSPERO, identifier [CRD42020208209].

## Introduction

Neuromyelitis optica (NMO) is a serious inflammatory demyelinating disease in the central nervous system (CNS), characterized by preferential lesions in the optic nerve and spinal cord (1–3). The term “spectrum of neuromyelitis optica” was first proposed by Wingerchuk in 2007, incorporating the condition recently known as NMO and patients with restricted lesions [e.g., longitudinal extensive transverse myelitis (LETM), isolated optic neuritis (ON), area postrema syndrome, and brainstem syndrome] (4).The diagnostic international consensus for neuromyelitis optica spectrum disorder (NMOSD) was published in 2015 (5). Both NMO and NMOSD are associated with the presence of aquaporin 4-immunoglobulin G (AQP4-IgG) (6, 7), which was initially discovered in 2004 and existed on approximately 80–90% of patients with NMO and more than half of patients with NMOSD (8, 9). Aquaporin 4-immunoglobulin G selectively targets at aquaporin 4, the predominant water channel protein abundantly expressed in astrocytes, initiates complement-dependent cytotoxicity against astrocytes, leading to axonal, and myelin impairment (10, 11). Based on these pathogenic roles in NMO/NMOSD, AQP4-IgG became one of the required indicators in the 2006 NMO diagnostic criteria and the 2015 international consensus for NMOSD (1, 5). Aquaporin 4-immunoglobulin G can be detected in both the serum and cerebrospinal fluid (CSF), while there have been reports demonstrating that detection of AQP4-IgG is less sensitive in the CSF than in the serum, especially in the remission phase of the disease (12, 13). Therefore, with the consideration that blood-derived AQP4-IgG is an easily accessible biomarker with high sensitivity, the serum is widely accepted to be an optimal and cost-effective choice for AQP4-IgG detection (13). To date, the clinical significance of monitoring serum AQP4-IgG titer in estimating relapse and assessing neurological impairment remains inconclusive. Due to the pathogenic role of AQP4-IgG in the NMO/NMOSD and since most AQP4-IgG is produced in the peripheral lymphoid tissue, clinical relapse is assumed to be associated with an increase in serum AQP4-IgG titer, and this is thought to cause more serious damage to the CNS (13, 14). These demonstrations have been supported by several clinical observations, revealing the link between serum AQP4-IgG titers and clinical episodes or parameters including clinical attack or acute exacerbation, neurological disability evaluated by the Expanded Disability Status Scale (EDSS), and long vertebral segment lesions in the spinal cord (15–21). However, there have been a number of studies indicating that serum AQP4-IgG titer could not reflect disease activity, severity, or neurological prognosis, except for its usefulness in NMO/NMOSD diagnosis (12, 22–26). Therefore, to evaluate the clinical relevance and clinical utility of serum AQP4-IgG titer monitoring, we performed a systematic review and meta-analysis to assess the association between serum AQP4-IgG titers and NMOSD activity and severity.

## Methods

### Objective

We sought to determine (1) whether the serum AQP4-IgG titer of patients with NMO/NMOSD in the attack phase is significantly different from that of patients in the remission phase and (2) whether the serum AQP4-IgG titer of patients with NMO/NMOSD is correlated with the clinical parameters in the attack phase, including the EDSS score and number of involved spinal cord segments. The meta-analysis was registered on PROSPERO (ID CRD42020208209) and conducted according to the predetermined protocols involving the Meta-analysis of Observational Studies in Epidemiology Recommendations for Study Reporting and the Preferred Reporting Items for Systematic Reviews and Meta-analysis (PRISMA) statement.

### Literature Searching

Electronic databases including PubMed, Web of Science, Cochrane Library, and Embase were searched using the following terms: “Demyelinating Autoimmune Diseases, CNS,” “Myelitis, Transverse”, “Neuromyelitis Optica,” “Optic Neuritis,” “Neuromyelitis optica spectrum disorder,” “NMOSD,” “NMO,” “Disease, Devic,” “Syndrome, Devic,” “Aquaporin 4,” “Aquaporin 4 IgG,” “AQP4 IgG,” “Aquaporin 4 antibody,” “AQP4 antibody,” “AQP4-Ab,” “Antibodies to AQP4,” “NMO-IgG,” and “AQP4”. “Neuromyelitis optica,” “Demyelinating Autoimmune Diseases, CNS,” “Myelitis, Transverse,” “Optic Neuritis,” and “Aquaporin 4” were used as the MeSH terms and keywords; other terms were searched as keywords. No publication type restrictions were imposed. Publications, which were not written in English or Chinese, were excluded. The literature search date was from January 1, 2004 to March 3, 2020. Two authors (JL and KS) independently conducted the literature search and data extraction with the same standardized method, and any inconsistencies were resolved by discussion among all authors.

### Selection (Inclusion and Exclusion) Criteria

Studies were included if they were original research comparing the serum AQP4-IgG titer of patients with NMO/NMOSD between the attack phase and remission phase or evaluating the correlation between serum AQP4-IgG titer and clinical parameters including the EDSS score and number of involved spinal cord segments in the attack phase. Patients of any age were included, with no restrictions on disease duration, disability, comorbidities, or treatment. All detection methods for AQP4-IgG were also included. Diagnosis of NMO or NMOSD should be stated with reference to the published diagnostic criteria or international consensus. Studies were excluded if they were reviews, unrelated to the research topic, or basic science or animal studies. When data of the patients overlapped in several articles, only the one with the most complete information was included. Studies could also be retrospective or prospective. Detailed information on the selection criteria is provided in Supplementary Tables 1, 2.

### Data Extraction

For the meta-analysis of comparing the serum AQP4-IgG titer of patients with NMO or NMOSD between the attack and remission phase, the following data were extracted: the first author, publication year, diagnosis and diagnostic criteria, methods used for serum AQP4-IgG detection, serum AQP4-IgG titer, number of patients and serum samples, and demographic characteristics including age at sampling, sex ratio, and ethnicity. Data were reported as mean and standard deviation (SD), median and range/interquartile range (IQR), or a figure (without quantitative value). If data were not provided as mean and SD, the authors were contacted to provide the value of mean and SD or the raw data of the AQP4-IgG titer. For the correlation meta-analysis, the following data were extracted: the first author, publication year, diagnosis and diagnostic criteria, methods used for serum AQP4-IgG detection, number of patients and serum samples, demographic characteristics including age at sampling and ethnicity, and the Pearson or Spearman correlation coefficient (COR) value. The authors of eight studies did not respond to our data request, and the mean and SD were estimated for five (median and range/IQR were available in these studies) of them using the methods reported by Luo et al. and Wan et al., respectively (27, 28). Finally, 14 studies were included in the meta-analysis (11 studies were included in the meta-analysis of comparing serum AQP4-IgG titer between the attack and remission phase, and seven studies were included in the correlation meta-analysis). The Newcastle–Ottawa Scale (NOS), a scoring system for quality assessment of non-randomized research, was utilized for the included studies.

### Statistical Analysis

Meta-analysis was performed using R (version 3.5.1). For the continuous variables, the standard mean difference (SMD) with a 95% confidence interval (95% CI) was used. The heterogeneity among studies was tested by calculating Q statistic and inconsistency index (*I*^2^). A random-effects model was used to minimize the impact of the heterogeneity. The stability of results and the source of heterogeneity were evaluated by the sensitivity analysis and meta-regression, respectively; if necessary, further subgroup analysis was performed. The Spearman COR value was converted to the Pearson COR value before z-transformation according to the methods reported by Rupinski et al. (29). The COR values were converted by Fisher's r-to-z transformation to obtain the approximate z-values of the normal distribution to further calculate the 95% CI. Pooled CORs were transformed back to the raw scale for presentation. Publication bias was assessed using the Egger test.

## Results

### Description of Studies

**Figure 1:**
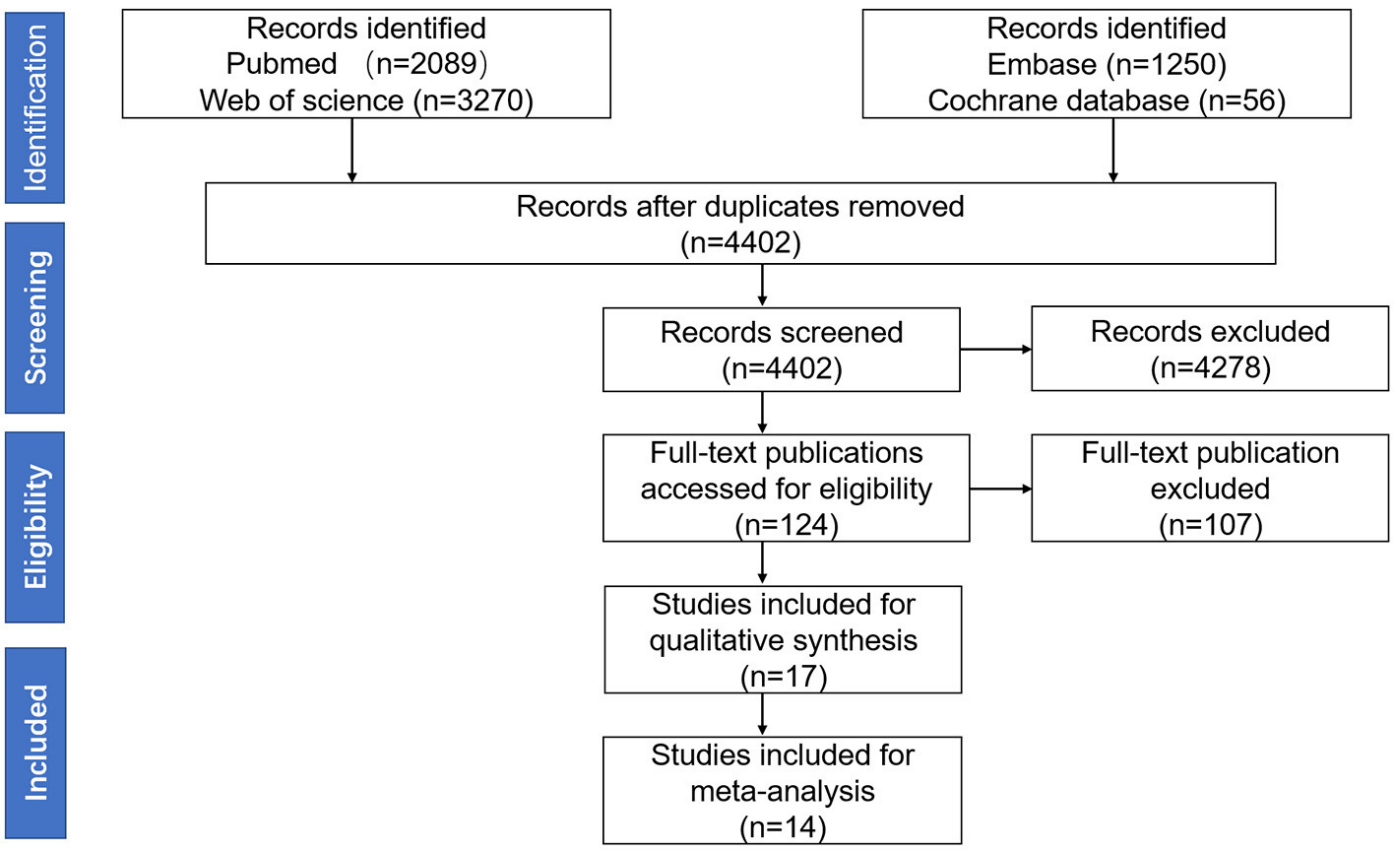
The Preferred Reporting Items for Systematic Reviews and Meta-analysis (PRISMA) 2020 flow diagram of publication identification.

A total of 14 studies were included in this meta-analysis, among which 11 studies were included in the meta-analysis of comparing the serum AQP4-IgG titer between the attack and remission phase (224 serum samples in the attack phase and 536 serum samples in the remission phase), three studies were included in the correlation meta-analysis between the serum AQP4-IgG titer and EDSS score (92 serum samples) in the attack phase, and four studies were included in the correlation meta-analysis between the serum AQP4-IgG titer and number of involved spinal cord segments (59 serum samples) in the attack phase. All the serum samples were from patients diagnosed as NMO or NMOSD, which fulfilled the diagnostic criteria or international consensus for NMO or NMOSD published in the year 1999, 2006, 2007, or 2015 (1, 3–5). There were three kinds of methods for serum AQP4-IgG detection: cell-based assay [CBA, CBA-based indirect immunofluorescence (CBA-IFI), CBA-recombinant immunofluorescence assay (CBA-rIFA), and CBA-based fluorescence-activated cell sorting (CBA-FACS)], enzyme-linked immunosorbent assay (ELISA), and fluorescence-based immunoprecipitation assay (FIPA). In general, the AQP4-IgG positivity rate of the patients or samples included in all 11 studies of comparison of serum AQP4-IgG titer between the attack and remission phase was 100% (Supplementary Table 3). Enrolled patients were from different countries, including China, Japan, Hungary, Germany, Austria, Italy, and other unspecified countries. The flow diagram of publication identification is shown in Figure 1. The demographic and clinical characteristics of patients in the included studies are shown in Tables 1–3. The quality evaluation of the studies is presented in Table 4, and the PRISMA checklist is in Supplementary Table 4. In addition, there was no obvious publication bias across studies assessed using the Egger test (*p* = 0.31).

**Table 1:** Studies included in the meta-analysis for comparison of serum aquaporin 4-immunoglobulin G (AQP4-IgG) titer between the attack and remission phase.

								***Attack phase***		***Remission phase***	
**Author, year (Reference)**	**No. patients**	**Age**	**Gender (F:M)**	**Ethnicity**	**AQP4-IgG test method**	**Diagnosis**	**Diagnostic criteria**	**No. patients/no. of serums**	**Tx**	**No. patients/no. of serums**	**Tx**
Huang, 2018 (19)	22	na	19:3	Chinese	CBA-IFI	NMOSD	2015^a^	na/19	na	na/17	na
Valentino, 2017 (30)	7	38.9^+^	6:1	na	CBA-IFI	NMO	2006^b^	5/10	None	7/261	RTX
Majed, 2016 (13)	na	na	na	na	CBA-FACS	NMO/NMOSD	2006^b^/2007^c^	na/8	na	na/15	na
Liang, 2019 (26)	136	na	120:16	Chinese	CBA-IFI	NMOSD	2015^a^	97/65	na	39/26	na
Kovacs, 2016 (20)	6	na	6:0	Hungarian	CBA-FACS	NMO	2006^b^	4/4	na	6/15	na
Jarius, 2010 (12)	37	41^++^	17.5:1	German, Austrian, and Italian	CBA-rIFA	NMO/NMOSD	2006^b^/2007^c^	29/20	na	11/6	na
Jarius, 2008 (16)	8	45^++^	7:1	German	FIPA	NMO/NMOSD	2006^b^/2007^c^	8/20	na	8/57	na
Isobe, 2013 (25)	38	na	na	Japanese	ELISA	NMO/NMOSD	2006^b^/2007^c^	12/20	None	12/17	IS
Isobe, 2012 (29)	29	49.5^+^	26:3	Japanese	ELISA	NMO	1999^d^	9/28	None	9/79	na
Chanson, 2013 (31)	10	44.3^+^	10:0	na	CBA-FACS	NMO	2006^b^	na/10	None	na/20	IS
Akaishi, 2020 (24)	45	44.3^+^	44:1	Japanese	CBA-IFI	NMOSD	2015^a^	16/23	None	16/23	IS

*AQP4, aquaporin4; CBA, cell-based assay; FACS, luorescence-activated cell sorting; ELISA, enzyme linked immunosorbent assay; F:M, female:male; IFI, indirect immunofluorescence; FIPA, fluorescence-based immunoprecipitation assay; IS, immunosuppressant; na, not available; NMO, neuromyelitis optica; NMOSD, neuromyelitis optica spectrum disorder; rIFA, recombinant immunofluorescence assay; RTX, Rituximab; Tx, treatment*.

+ *Indicates mean value*;

++ *Indicates median value*.

a *Wingerchuk et al. (5)*.

b *Wingerchuk et al. (1)*.

c *Wingerchuk et al. (4)*.

d *Wingerchuk et al. (3)*.

**Table 2:** Studies included in the meta-analysis for the correlation between the serum AQP4-IgG titer and the number of involved spinal cord segments.

**First author, year**	**No. of patients**	**Age**	**Ethnicity**	**AQP4-IgG test method**	**Diagnosis**	**Diagnostic criteria**	**Tx**	***n***	***r***
Takahashi, 2007 (15)	NMO = 22; HR-NMO = 13	41^++^(NMO); 37^++^ (HR-NMO)	Japanese	CBA-IFI	NMO/HR-NMO	2006^a^	None	20	0.91^*^
Isobe, 2012 (29)	29	49.3^+^	Japanese	CBA-FACS	NMO	1999^b^	None	18	0.35^**^
Dujmovic, 2011 (22)	12	49^++^	na	CBA-IFI	NMO/HR-NMO	2006^a^	None = 3; CS = 5; CS ^+^ IS = 2; CS ^+^ PE ^+^ IS = 2	12	0.68^**^
Li, 2013 (21)	16	41^++^	Chinese	CBA-IFI	NMO	2006^a^	None	9	0.55^*^

*AQP4, aquaporin4; CBA, cell-based assay; CS, corticosteroids; FACS, fluorescence-activated cell sorting; IFI, indirect immunofluorescence; IS, immunosuppressants (cyclophosphamide or azathioprine); PE, plasma exchange; NMO, neuromyelitis optica; HR-NMO, high risk of NMO; n, number of samples; na, not available; r, correlation coefficient value; Tx, treatment*.

+ *Indicates mean value*.

++ *Indicates median value*.

* *Indicates Pearson correlation coefficient value*.

** *Indicates Spearman correlation coefficient value*.

a *Wingerchuk et al. (1)*.

b *Wingerchuk et al. (3)*.

**Table 3:** Studies included in the meta-analysis for the correlation between the serum AQP4-IgG titer and the Expanded Disability Status Scale (EDSS) score.

**First author, year**	**No. of patients**	**Age**	**Ethnicity**	**AQP4-IgG test method**	**Diagnosis**	**Diagnostic criteria**	**Tx**	***n***	***r***
Liang, 2019 (26)	97	42^+^	Chinese	CBA-IFI	NMOSD	2015^a^	na	65	0.30^**^
Li, 2013 (21)	16	41^++^	Chinese	CBA-IFI	NMO	2006^b^	None	9	0.90^*^
Isobe, 2012 (29)	29	49.3^+^	Japanese	CBA-FACS	NMO	1999^c^	None	18	0.31^**^

*AQP4, aquaporin4; CBA, cell-based assay; EDSS, Expanded Disability Status Scale; FACS, fluorescence-activated cell sorting; IFI, indirect immunofluorescence; NMO, neuromyelitis optica; NMOSD, neuromyelitis optica spectrum disorder; n, number of samples; na, not available; r, correlation coefficient value; Tx, treatment*.

+ *Indicates mean value*;

++ *Indicates median value*.

* *Indicates Pearson correlation coefficient value*.

** *Indicates Spearman correlation coefficient value*.

a *Wingerchuk et al., (5)*.

b *Wingerchuk et al. (1)*.

c *Wingerchuk et al. (3)*.

**Table 4:** Quality evaluation of the included studies for the meta-analysis.

	**Quality (NOS)**		
**Study**	Selection (4*)	Comparability (3*)	Exposure (2*)
Huang et al. (19)	****		**
Valentino, 2017 (30)	***	**	**
Majed et al. (13)	**		**
Liang et al. (26)	****	*	**
Kovacs et al. (20)	****		**
Jarius et al. (12)	***		**
Jarius et al. (16)	***		**
Isobe et al. (25)	***		**
Isobe et al. (29)	****		**
Chanson (31)	****	**	**
Akaishi et al. (24)	**	*	**
Takahashi et al. (15)	***	*	**
Dujmovic et al. (22)	***	*	**
Li et al. (21)	***		**

Each study is scored using a star system based on three domains: (1) selection of study groups—maximum 4*; (2) comparability of the groups—maximum 2*; (3) ascertainment of outcome—maximum 3*. NOS, Newcastle–Ottawa Scale.

### Comparison of Serum Aquaporin 4-Immunoglobulin G Titer Between the Attack and Remission Phase

**Figure 2:**
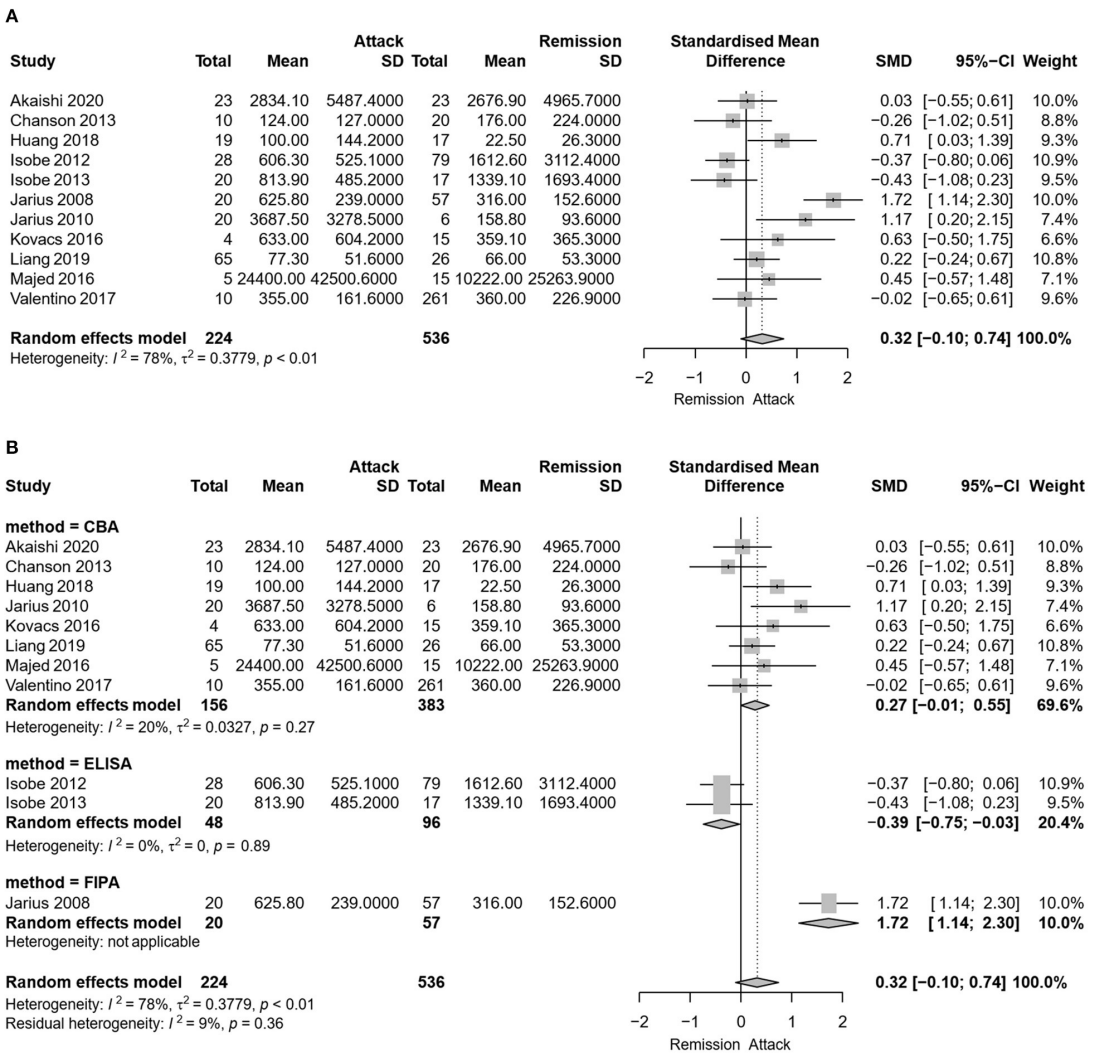
Meta-analysis of the serum aquaporin 4-immunoglobulin G (AQP4-IgG) titer between the attack and remission phase for patients with neuromyelitis optica spectrum disorder (NMOSD). **(A)** Meta-analysis of the serum AQP4-IgG titer between the attack and remission phase for patients with NMOSD. **(B)** Subgroup meta-analysis of the serum AQP4-IgG titer between the attack and remission phase for patients with NMOSD according to the antibody detection methods. AQP4-IgG, aquaporin4-IgG; CBA, cell-based assay; ELISA, enzyme linked immunosorbent assay; FIPA, fluorescence-based immunoprecipitation assay; NMOSD, neuromyelitis optica spectrum disorder; SD, standard deviation; 95% CI, 95% confidence interval.

Eleven studies, including 224 serum samples in the attack phase and 536 serum samples in the remission phase, were included in the meta-analysis of comparing the serum AQP4-IgG titer between the attack and remission phase. Heterogeneity assessed by *I*^2^ demonstrated an obvious variety among studies (*I*^2^ = 78%), and the results showed that there was no significant difference in serum AQP4-IgG titer between the attack and remission phase in patients with NMO or NMOSD [SMD: 0.32, 95% CI (−0.10, 0.74), *p* = 0.14] (Figure 2A).

**Figure 3:**
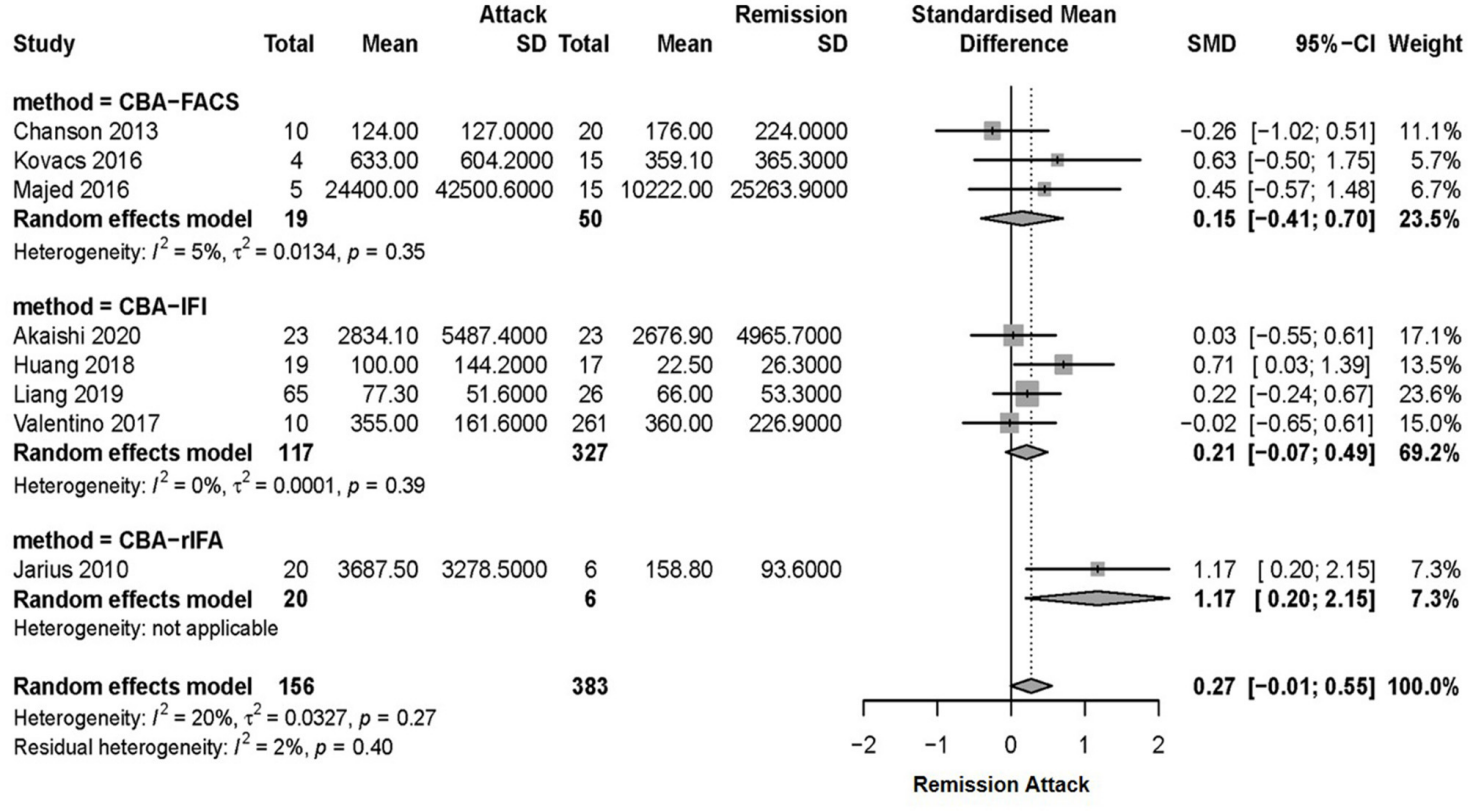
Subgroup meta-analysis of serum AQP4-IgG titer tested by CBA between the attack and remission phase for patients with NMOSD. AQP4-IgG, aquaporin4-IgG; CBA, cell-based assay; FACS, fluorescence-activated cell sorting; IFI, indirect immunofluorescence; NMOSD, neuromyelitis optica spectrum disorder; SD, standard deviation; rIFA, recombinant immunofluorescence assay; 95% CI, 95% confidence interval.

Due to the heterogeneity among studies, sensitivity analysis was conducted and confirmed the stability of the results (Supplementary Figure 1). Meta-regression analysis was conducted to evaluate the potential source of the heterogeneity (i.e., ethnicity, antibody detection method, and diagnosis). Medication at sampling was not evaluated in the meta-regression analysis, since there were only five studies in the attack phase and four studies in the remission phase that reported detailed medication information for patients. According to the meta-regression analysis results, the heterogeneity depended on the antibody detection method (*p* = 0.007), rather than different ethnicity (*p* > 0.05) or diagnosis of NMO/NMOSD (*p* > 0.05). Therefore, a subgroup meta-analysis according to the serum AQP4-IgG detection method was performed (Figure 2B). Among the 11 included studies, eight studies used CBA, two studies used ELISA, and one study used FIPA for serum AQP4-IgG detection. The results showed that the heterogeneity assessed by *I*^2^ was significantly decreased in the subgroup analysis (${\text{I}}_{\text{CBA}}^{2}$ = 20%, ${\text{I}}_{\text{ELISA}}^{2}$ = 0%) and there was also no statistically significant difference in serum AQP4-IgG titer between the attack and remission phase in the subgroup analysis of serum AQP4-IgG detected by CBA [SMD: 0.27, 95% CI (−0.01, 0.55), *p* = 0.06]. There was a significantly higher serum AQP4-IgG titer in the remission phase compared with that in the attack phase in the subgroup analysis of serum AQP4-IgG detected by ELISA [SMD: −0.39, 95% CI (−0.75, −0.03), *p* = 0.04]. Moreover, meta-analysis of comparing the serum AQP4-IgG titer between the attack and remission phase was further performed for the cases in which serum AQP4-IgG were detected by different CBA methods (four studies used CBA-IFI, one study used CBA-rIFA, and three studies used CBA-FACS for serum AQP4-IgG detection). The heterogeneity of the studies with CBA-IFI and CBA-FACS assessed by *I*^2^ demonstrated no obvious variety among them (${\text{I}}_{\text{CBA}-\text{IFI}}^{2}$ = 0%, ${\text{I}}_{\text{CBA}-\text{FACS}}^{2}$ = 5%), and the results showed that there was no significant difference in serum AQP4-IgG titer between the attack and remission phase in each of the subgroup analysis above [CBA-IFI: SMD: 0.21, 95% CI (−0.07, 0.49), *p* = 0.15; CBA-FACS: SMD: 0.15, 95% CI (−0.41, 0.70), *p* = 0.60] (Figure 3).

Additionally, among the 11 studies, there were three studies [Jarius et al. (16), Akaishi et al. (24), and Isobe et al. (29)] that included attack–remission paired serum samples (45 samples) from the same NMOSD patient, illustrating no significant difference in serum AQP4-IgG titer between the attack and remission phase in the two [Akaishi et al. (24), Isobe et al. (29)], while one indicated significantly increased serum AQP4-IgG titer in the attack phase than in the remission phase [Jarius et al. (16); the quantitative value of AQP4-IgG titer was unavailable].

### Correlation Between the Serum Aquaporin 4-Immunoglobuling Titer and the Number of Involved Spinal Cord Segments in the Attack Phase

**Figure 4:**
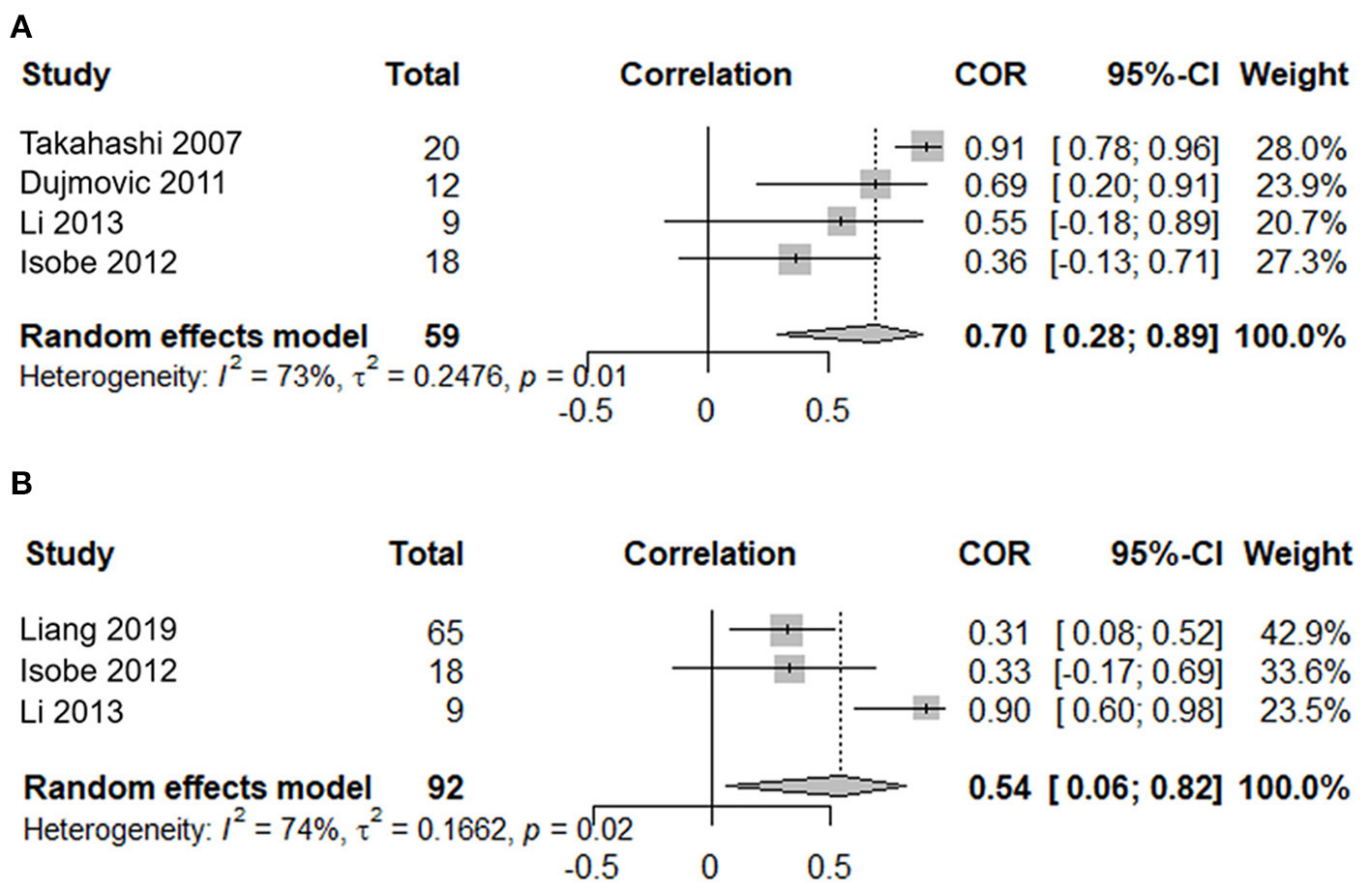
Correlation meta-analysis between the serum AQP4-IgG titer and clinical parameters in the attack phase for patients with NMOSD. **(A)** Correlation meta-analysis between the serum AQP4-IgG titer and the number of involved spinal cord segments. **(B)** Correlation meta-analysis between the serum AQP4-IgG titer and the EDSS score. AQP4-IgG, aquaporin4-IgG; NMOSD, neuromyelitis optica spectrum disorder; COR, correlation coefficients; EDSS, expanded disability status scale; 95% CI, 95% confidence interval.

Four studies, including 59 serum samples in the attack phase, were used in the meta-analysis for the correlation between the serum AQP4-IgG titer and the number of involved spinal cord segments. Heterogeneity, assessed by *I*^2^, demonstrated obvious variation among studies (*I*^2^ = 73%), and the random effects model was used. The results showed that the serum AQP4-IgG titer was positively correlated with the number of involved spinal cord segments in the attack phase [COR 0.70, 95% CI (0.28–0.89), *p* = 0.003] (Figure 4A). Sensitivity analysis confirmed the stability of this result.

### Correlation Between the Serum Aquaporin 4-Immunoglobulin G Titer and the Expanded Disability Status Scale in the Attack Phase

Three studies, including 92 serum samples in the attack phase, were included in the meta-analysis for the correlation between the serum AQP4-IgG titer and the EDSS. Heterogeneity, assessed by *I*^2^, demonstrated obvious variation among studies (*I*^2^ = 74%), and the random effects model was used. The results showed that the serum AQP4-IgG titer was positively correlated with the EDSS score in the attack phase [COR 0.54, 95% CI (0.06–0.82), *p* = 0.03] (Figure 4B). Sensitivity analysis confirmed the stability of this result.

## Discussion

The importance of AQP4-IgG as a diagnostic biomarker in NMO or NMOSD has been established for years (1, 5). However, the clinical relevance of this antibody in assessing the disease activity or severity has not yet been determined. Compared with the invasive lumbar puncture for CSF collection, serological specimens are more convenient and easily accessible, which make AQP4-IgG detection in serum an ideal approach in the clinical practice for AQP4-IgG titer monitoring (13).

In the present study, we performed a systematic review and meta-analysis of the clinical relevance of serum AQP4-IgG titer. This not only assessed whether the level of this antibody was associated with disease activity by meta-analysis of serum AQP4-IgG titer between the attack and remission phase but also evaluated whether its level was correlated with disease severity by correlation meta-analysis between the serum AQP4-IgG titer and the number of involved spinal cord segments or the EDSS score in the attack phase. The results demonstrated that there was no significant difference in serum AQP4-IgG titer between the attack and remission phase. However, due to the different serum AQP4-IgG detection methods applied in the included studies, obvious heterogeneity was observed. Therefore, we further performed a subgroup analysis according to the antibody detection methods. Among 11 included studies, serum AQP4-IgG was detected using CBA methods (CBA-IFI, CBA-rIFA, and CBA-FACS) in 8, and this method is currently the most widely used antibody detection method due to its high specificity and sensitivity. As a result, the heterogeneity of this subgroup analysis assessed by *I*^2^ (including studies in which AQP4-IgG was detected by CBA) was evidently decreased to 20%. The result of this subgroup meta-analysis was consistent with the general one including all 11 studies. These findings suggest that a standard antibody detection method would lead to more homogeneous antibody detection results among different studies, although the different demographic or clinical features in enrolled patients and somewhat different detailed experimental procedures, which calls for the attempts to standardize AQP4-IgG assays among different laboratories. There were only three studies [Jarius et al. (16), Akaishi et al. (24), and Isobe et al. (29)] that included attack–remission paired serum samples from the same patient. One of them did not provide the quantitative value of the serum AQP4-IgG titer (16); therefore, a meta-analysis comparing the antibody levels between the attack and remission phase with these paired samples was not possible in the present study. Consequently, although the definite pathogenicity of AQP4-IgG in NMOSD was established, there was no significant difference in serum AQP4-IgG titer between the relapse and remission phase in patients with NMOSD, suggesting that serum AQP4-IgG titer was not associated with disease activity in this disorder, and physicians may not be able to judge clinical attacks according to the serum AQP4-IgG titer. Therefore, further investigations to identify other novel biomarkers which can aid in the judgment of clinical episodes in NMOSD will be of great clinical significance.

In the correlation meta-analysis between the serum AQP4-IgG titer and the number of involved spinal cord segments or EDSS score in the attack phase, the results showed that the serum AQP4-IgG titer was positively correlated with both of these clinical parameters, suggesting that serum antibody titer could indicate the neurological impairment or disease severity in NMOSD during a clinical attack. These findings were also consistent with an animal experiment showing that astrocyte toxicity and axonal damage was associated with AQP4 antibody titer in mice and further confirmed the pathogenic role of AQP4-IgG in NMOSD (32). However, the significant heterogeneity, which may be due to the limited number of included studies in this correlative meta-analysis, suggests that these results should be considered with caution. Although the AQP4-IgG titer could indicate the disease severity in the attack phase, it was not associated with the disease activity in this study, which may be due to the following factors: (1) The current study showed that serum AQP4-IgG titer was positively correlated with the attack severity, indicating that patients with mild attacks had lower levels of AQP4-IgG than those with severe attacks. In the studies that compared the AQP4-IgG titer between the attack and remission phase, there were no detailed descriptions regarding the attack severity, and if a certain number of patients with mild attacks and lower levels of AQP4-IgG were included, the overall levels of AQP4-IgG would be comparatively lower, leading to no significant difference in antibody levels between the attack and remission phase. (2) Studies included in the meta-analysis of comparing the serum AQP4-IgG titer between the attack and remission phase lacked enough attack–remission paired samples from the same patient. It is known that the individual differences in serum antibody level between patients are quite obvious, and several patients can have comparatively higher levels of antibodies even in the remission phase. If the serum of these patients were included only in the remission phase but not in the attack phase, it would increase the overall antibody level in the remission phase and affect the results of this comparison to some extent.

There were some limitations in the present study. First, due to the rarity of NMOSD, the number of patients enrolled in each study included in this meta-analysis was limited. The number of studies that investigated the difference of serum AQP4-IgG titer between the attack and remission phase (especially with an attack-remission paired sample from the same patient) was also limited. Second, the AQP4-IgG detection methods were different among studies, leading to the obvious heterogeneity in results among studies, although CBA had been widely applied in recent years. Third, medication information at serum sampling was limited (available in only five studies in the attack phase and four studies in the remission phase). This is of importance, as it is known that different treatments may influence serum AQP4-IgG titer. Therefore, the conclusions of the present study are very preliminary and need to be considered with caution. A further meta-analysis with a larger number of studies that employed standardized AQP4-IgG assays and detected attack–remission paired samples from the same patients with detailed medication information will be required to confirm our findings and further optimize the clinical practice of serum AQP4-IgG monitoring.

## Conclusions

The present systematic review and meta-analysis provided the first comprehensive evaluation of the clinical relevance of serum AQP4-IgG titer in patients with NMOSD, indicating that the serum AQP4-IgG titer was not associated with the disease activity but indicated the disease severity in the attack phase in NMOSD.

## Data Availability Statement

The raw data supporting the conclusions of this article will be made available by the authors, without undue reservation.

## Author Contributions

JL study concept and design, acquisition and interpretation of data, and drafting. GT and BL critical revision of manuscript. JZ, YG, and YC revision of manuscript. ZJ study design, critical revision of manuscript. KS study design, acquisition and interpretation of data, and critical revision of manuscript.

## Funding

This work was supported by the National Natural Science Foundation of China (Grant No. 81904131), the Chinese Medicine Inheritance and Innovation Talent Project—leading talent support program of the National Traditional Chinese Medicine (Grant No. 2018, No. 12), and the Beijing University of Chinese Medicine project (Grant No. 2020-tsxk-001).

## Conflict of Interest

The authors declare that the research was conducted in the absence of any commercial or financial relationships that could be construed as a potential conflict of interest.

## Publisher's Note

All claims expressed in this article are solely those of the authors and do not necessarily represent those of their affiliated organizations, or those of the publisher, the editors and the reviewers. Any product that may be evaluated in this article, or claim that may be made by its manufacturer, is not guaranteed or endorsed by the publisher.

## References

[1] Wingerchuk DM Lennon VA Pittock SJ Lucchinetti CF Weinshenker BG. Revised diagnostic criteria for neuromyelitis optica. Neurology. (2006) 66:1485–9. 10.1212/01.wnl.0000216139.44259.74 16717206

[2] de Seze J Stojkovic T Ferriby D Gauvrit JY Montagne C Mounier-Vehier F . Devic's neuromyelitis optica: clinical, laboratory, MRI and outcome profile. J Neurol Sci. (2002) 197:57–61. 10.1016/S0022-510X(02)00043-6 11997067

[3] Wingerchuk DM Hogancamp WF O'Brien PC Weinshenker BG. The clinical course of neuromyelitis optica (Devic's syndrome). Neurology. (1999) 53:1107–14. 10.1212/WNL.53.5.1107 10496275

[4] Wingerchuk DM Lennon VA Lucchinetti CF Pittock SJ Weinshenker BG. The spectrum of neuromyelitis optica. Lancet Neurol. (2007) 6:805–15. 10.1016/S1474-4422(07)70216-8 17706564

[5] Wingerchuk DM Banwell B Bennett JL Cabre P Carroll W Chitnis T . International consensus diagnostic criteria for neuromyelitis optica spectrum disorders. Neurology. (2015) 85:177–89. 10.1212/WNL.0000000000001729 26092914 PMC4515040

[6] Lennon VA Wingerchuk DM Kryzer TJ Pittock SJ Lucchinetti CF Fujihara K . A serum autoantibody marker of neuromyelitis optica: distinction from multiple sclerosis. Lancet. (2004) 364:2106–12. 10.1016/S0140-6736(04)17551-X 15589308

[7] Lennon VA Kryzer TJ Pittock SJ Verkman AS Hinson SR. IgG marker of optic-spinal multiple sclerosis binds to the aquaporin-4 water channel. J Exp Med. (2005) 202:473–7. 10.1084/jem.20050304 16087714 PMC2212860

[8] Sato DK Callegaro D Lana-Peixoto MA Waters PJ de Haidar Jorge FM Takahashi T . Distinction between MOG antibody-positive and AQP4 antibody-positive NMO spectrum disorders. Neurology. (2014) 82:474–81. 10.1212/WNL.0000000000000101 24415568 PMC3937859

[9] Cook LJ Rose JW Alvey JS Jolley AM Kuhn R Marron B . Collaborative international research in clinical and longitudinal experience study in NMOSD. Neurol Neuroimmunol Neuroinflamm. (2019) 6:e583. 10.1212/NXI.0000000000000583 31355319 PMC6624150

[10] Misu T Fujihara K Kakita A Konno H Nakamura M Watanabe S . Loss of aquaporin 4 in lesions of neuromyelitis optica: distinction from multiple sclerosis. Brain. (2007) 130:1224–34. 10.1093/brain/awm047 17405762

[11] Roemer SF Parisi JE Lennon VA Benarroch EE Lassmann H . Pattern-specific loss of aquaporin-4 immunoreactivity distinguishes neuromyelitis optica from multiple sclerosis. Brain. (2007) 130:1194–205. 10.1093/brain/awl371 17282996

[12] Jarius S Franciotta D Paul F Ruprecht K Bergamaschi R Rommer PS . Cerebrospinal fluid antibodies to aquaporin-4 in neuromyelitis optica and related disorders: frequency, origin, and diagnostic relevance. J Neuroinflammation. (2010) 7:52. 10.1186/1742-2094-7-52 20825655 PMC2945323

[13] Majed M Fryer JP McKeon A Lennon VA Pittock SJ. Clinical utility of testing AQP4-IgG in CSF: guidance for physicians. Neurol Neuroimmunol Neuroinflamm. (2016) 3:e231. 10.1212/NXI.0000000000000231 27144221 PMC4841640

[14] Verkman AS Phuan PW Asavapanumas N Tradtrantip L. Biology of AQP4 and anti-AQP4 antibody: therapeutic implications for NMO. Brain Pathol. (2013) 23:684–95. 10.1111/bpa.12085 24118484 PMC3890327

[15] Takahashi T Fujihara K Nakashima I Misu T Miyazawa I Nakamura M . Anti-aquaporin-4 antibody is involved in the pathogenesis of NMO: a study on antibody titre. Brain. (2007) 130:1235–43. 10.1093/brain/awm062 17449477

[16] Jarius S Aboul-Enein F Waters P Kuenz B Hauser A Berger T . Antibody to aquaporin-4 in the long-term course of neuromyelitis optica. Brain. (2008) 131:3072–80. 10.1093/brain/awn240 18945724 PMC2577801

[17] Waters P Jarius S Littleton E Leite MI Jacob S Gray B . Aquaporin-4 antibodies in neuromyelitis optica and longitudinally extensive transverse myelitis. Arch Neurol. (2008) 65:913–9. 10.1001/archneur.65.7.913 18625857

[18] Kim SH Kim SM Vincent A Ahn SW Hong YH Park KS . Clinical characteristics, prognosis, and seropositivity to the anti-aquaporin-4 antibody in Korean patients with longitudinally extensive transverse myelitis. J Neurol. (2010) 257:920–5. 10.1007/s00415-009-5438-2 20049472

[19] Huang X Xu FF Qian HR Lei X Qi XK Xia DY. [Clinical presentations of neuromyelitis optica spectrum disorders with ultra-longitudinally extensive transverse myelitis]. Zhonghua Yi Xue Za Zhi. (2018) 98:1658–63. 10.3760/cma.j.issn.0376-2491.2018.21.008 29925142

[20] Kovacs KT Kalluri SR Boza-Serrano A Deierborg T Csepany T Simo M . Change in autoantibody and cytokine responses during the evolution of neuromyelitis optica in patients with systemic lupus erythematosus: a preliminary study. Mult Scler. (2016) 22:1192–201. 10.1177/1352458515613165 26514978

[21] Li M Su W Wang J Pisani F Frigeri A Ma T. Detection of anti-aquaporin-4 autoantibodies in the sera of Chinese neuromyelitis optica patients. Neural Regen Res. (2013) 8:708–13. 10.3969/j.issn.1673-5374.2013.08.005 25206717 PMC4146080

[22] Dujmovic I Mader S Schanda K Deisenhammer F Stojsavljevic N Kostic J . Temporal dynamics of cerebrospinal fluid anti-aquaporin-4 antibodies in patients with neuromyelitis optica spectrum disorders. J Neuroimmunol. (2011) 234:124–30. 10.1016/j.jneuroim.2011.01.007 21316112

[23] Hinson SR McKeon A Fryer JP Apiwattanakul M Lennon VA Pittock SJ. Prediction of neuromyelitis optica attack severity by quantitation of complement-mediated injury to aquaporin-4-expressing cells. Arch Neurol. (2009) 66:1164–7. 10.1001/archneurol.2009.188 19752309

[24] Akaishi T Takahashi T Nakashima I Abe M Ishii T Aoki M . Repeated follow-up of AQP4-IgG titer by cell-based assay in neuromyelitis optica spectrum disorders (NMOSD). J Neurol Sci. (2020) 410:116671. 10.1016/j.jns.2020.116671 31927341

[25] Isobe N Yonekawa T Matsushita T Masaki K Yoshimura S Fichna J . Clinical relevance of serum aquaporin-4 antibody levels in neuromyelitis optica. Neurochem Res. (2013) 38:997–1001. 10.1007/s11064-013-1009-0 23456674

[26] Liang J Liu J Fan R Chen Z Chen X Tong J . Plasma homocysteine level is associated with the expanded disability status scale in neuromyelitis optica spectrum disorder. Neuroimmunomodulation. (2019) 26:258–64. 10.1159/000503426 31655825

[27] Luo D Wan X Liu J Tong T. Optimally estimating the sample mean from the sample size, median, mid-range, and/or mid-quartile range. Stat Methods Med Res. (2018) 27:1785–805. 10.1177/0962280216669183 27683581

[28] Wan X Wang W Liu J Tong T. Estimating the sample mean and standard deviation from the sample size, median, range and/or interquartile range. BMC Med Res Methodol. (2014) 14:135. 10.1186/1471-2288-14-135 25524443 PMC4383202

[29] Isobe N Yonekawa T Matsushita T Kawano Y Masaki K Yoshimura S . Quantitative assays for anti-aquaporin-4 antibody with subclass analysis in neuromyelitis optica. Mult Scler. (2012) 18:1541–51. 10.1177/1352458512443917 22526930

[30] Valentino P Marnetto F Granieri L Capobianco M Bertolotto A. Aquaporin-4 antibody titration in NMO patients treated with rituximab: a retrospective study. Neurol Neuroimmunol Neuroinflamm. (2017) 4:e317. 10.1212/NXI.0000000000000317 28054001 PMC5182057

[31] Chanson JB Alame M Collongues N Blanc F Fleury M Rudolf G . Evaluation of clinical interest of anti-aquaporin-4 autoantibody followup in neuromyelitis optica. Clin Dev Immunol. (2013) 2013:146219. 10.1155/2013/146219 23710199 PMC3655457

[32] Herwerth M Kalluri SR Srivastava R Kleele T Kenet S Illes Z . In vivo imaging reveals rapid astrocyte depletion and axon damage in a model of neuromyelitis optica-related pathology. Ann Neurol. (2016) 79:794–805. 10.1002/ana.24630 26946517 PMC5021140

