# Supplementary material for: Serum Aquaporin 4-Immunoglobulin G Titer and Neuromyelitis Optica Spectrum Disorder Activity and Severity: A Systematic Review and Meta-Analysis
Source: Front Neurol. 2021 Oct 20;12:746959. doi: 10.3389/fneur.2021.746959 (PMC8565925; doi:10.3389/fneur.2021.746959)
Supplement: Supplementary file 1 [file Data_Sheet_1.docx]

| **Supplemental Table 1. Inclusion / Exclusion Criteria** |  |
| --- | --- |
| **Inclusion Criteria** | **Exclusion Criteria** |
| Diagnosis of NMO or NMOSD with an established diagnostic  criteria or international consensus. | Inflammatory demyelinating diseases in the central nervous system could not fulfill the established diagnostic criteria of NMO/NMOSD. |
| Case control studies (retrospective or prospective)  of original work. | Animal studies, reviews or responses, or studies  unrelated to the research topic. |
| Serum AQP4-IgG was measured in a quantitative  manner. | The quantitative value of AQP4-IgG titer was unavailable. |
| Validated assay or, if not, description of detailed  technique protocols. | Language (Not English or Chinese). |

| **Supplemental Table 2. Reasons for exclusion** | | | | | | | | |
| --- | --- | --- | --- | --- | --- | --- | --- | --- |
|  | | | **Reason for exclusion** | | | **Number** | | |
| **Excluded on Title or Abstract** | | | Summary or review or only abstract was available. | | | 2155 | | |
|  |  |  | Basic science study or Animal (not clinical) study. | | | 807 | | |
|  |  |  | Not NMO or NMOSD. | | | 355 | | |
|  |  |  | Not related to the research topic. | | | 862 | | |
|  |  |  | Duplicated publications. | | | 99 | | |
|  |  |  | Total | | | 4278 | | |
| **Excluded on Full-text** | | | Full-text was not available. | | | 9 | | |
|  |  |  | Language (Not English or Chinese). | | | 3 | | |
|  |  |  | Not NMO or NMOSD. | | | 5 | | |
|  |  |  | The quantitative value of AQP4-IgG titer was unavailable. | | | 18 | | |
|  |  |  | Not related to the research topic. | | | 72 | | |
|  |  |  | Total | | | 107 | | |
| **Excluded as the required dataset was unavailable** | | | | | | 3 | | |
| **Included in the meta-analysis** | | | | | | 14 | | |
| **Supplemental Table 3. The expression and positivity rate of AQP4-IgG and the definitions of the relapse and remission phase in the studies included in the meta-analysis** | | | | | | | | |
| **Author, year** | | **AQP4-IgG test method** | **AQP4-IgG titer expression** | | **Positivity rate of AQP4-IgG** | **Relapse phase** | | **Remission phase** |
| Huang, 2018 | | CBA-IFI | Serum dilution factor | | 100%** | Within 2 weeks after attack onset | | >30 days after attack onset |
| Valentino, 2017 | | CBA-IFI | Serum dilution factor | | 100%* | Within 30 days after attack onset | | 2–6 months after relapse or 4–6 months preceding relapse |
| Majed, 2016 | | CBA-FACS | Serum dilution factor | | 100%** | Within 30 days after attack onset | | >30 days after attack onset |
| Liang, 2019 | | CBA-IFI | Serum dilution factor | | 100%* | na | | na |
| Kovacs, 2016 | | CBA-FACS | Median fluorescence intensities (MFI) | | 100%* | na | | na |
| Jarius, 2010 | | CBA-rIFA | Serum dilution factor | | 100%*** | Within 30 days from onset of the most recent attack | | >30 days after attack onset |
| Jarius, 2008 | | FIPA | Arbitrary fluorescence units (FU) | | 100%* | na | | na |
| Isobe, 2013 | | ELISA | Units (U)/mL | | 100%* | Within 30 days after the initiation of attack | | At least 30 days after the previous relapse and at least 100 days before any subsequent relapse |
| Isobe, 2012 | | ELISA | Units (U)/mL | | 100%* | Within 30 days after the initiation of attack | | At least 30 days after the previous relapse and at least 100 days before any subsequent relapse |
| Chanson, 2013 | | CBA-FACS | Arbitrary units (AU)/mL | | 100%* | Within 30 days after attack onset | | >30 days after attack onset |
| Akaishi, 2020 | | CBA-IFI | Serum dilution factor | | 100%* | <1 month from the preceding clinical episode | | >3 months from the last attack |

AQP4, Aquaporin4; CBA, cell-based assay; FACS, Fluorescence-activated cell sorting; ELISA, enzyme linked immunosorbent assay; IFI, Indirect

immunofluorescence; FIPA, fluorescence-based immunoprecipitation assay; na, not available; rIFA, Recombinant immunoﬂuorescence assay.

* indicated patients with seropositive for AQP4-IgG were enrolled in the whole study.

**indicated only patients with seropositive for AQP4-IgG were included in the comparison of the AQP4-IgG titer between the attack and remission phase.

***indicated only serum samples with positive status of AQP4-IgG were included in the comparison of the AQP4-IgG titer between the attack and remission phase.

The attack was defined as patient-reported or objectively observed events typical of an acute inflammatory demyelinating event in the CNS, with a duration of at least 24 hours, in the absence of fever or infection, documented by contemporaneous neurologic examination.

| **Supplemental Table 4. PRISMA 2020 Checklist** | | | | | |
| --- | --- | --- | --- | --- | --- |
| **Section/topic** | | **#** | | **Checklist item** | **Reported on page #** |
| **TITLE** | | | | |  |
| **Title** | | 1 | | Identify the report as a systematic review, meta-analysis, or both. | 1 |
| **ABSTRACT** | | | | |  |
| **Structured summary** | | 2 | | Provide a structured summary including, as applicable: background; objectives; data sources; study eligibility criteria, participants, and interventions; study appraisal and synthesis methods; results; limitations; conclusions and implications of key findings; systematic review registration number. | 3-4 |
| **INTRODUCTION** | | | | |  |
| **Rationale** | | 3 | | Describe the rationale for the review in the context of what is already known. | 5-6 |
| **Objectives** | | 4 | | Provide an explicit statement of questions being addressed with reference to participants, interventions, comparisons, outcomes, and study design (PICOS). | 6-7 |
| **METHODS** | | | | |  |
| **Protocol and registration** | | 5 | | Indicate if a review protocol exists, if and where it can be accessed (e.g., Web address), and, if available, provide registration information including registration number. | 7 |
| **Eligibility criteria** | | 6 | | Specify study characteristics (e.g., PICOS, length of follow-up) and report characteristics (e.g., years considered, language, publication status) used as criteria for eligibility, giving rationale. | 7-8 |
| **Information sources** | | 7 | | Describe all information sources (e.g., databases with dates of coverage, contact with study authors to identify additional studies) in the search and date last searched. | 8-9 |
| **Search** | | 8 | | Present full electronic search strategy for at least one database, including any limits used, such that it could be repeated. | 7 |
| **Study selection** | | 9 | | State the process for selecting studies (i.e., screening, eligibility, included in systematic review, and, if applicable, included in the meta-analysis). | 7-8 |
| **Data collection process** | | 10 | | Describe method of data extraction from reports (e.g., piloted forms, independently, in duplicate) and any processes for obtaining and confirming data from investigators. | 8-9 |
| **Data items** | | 11 | | List and define all variables for which data were sought (e.g., PICOS, funding sources) and any assumptions and simplifications made. | 8-9 |
| **Risk of bias in individual studies** | | 12 | | Describe methods used for assessing risk of bias of individual studies (including specification of whether this was done at the study or outcome level), and how this information is to be used in any data synthesis. | 9-10 |
| **Summary measures** | | 13 | | State the principal summary measures (e.g., risk ratio, difference in means). | 9-10 |
| **Synthesis of results** | | 14 | | Describe the methods of handling data and combining results of studies, if done, including measures of consistency (e.g., I^2^) for each meta-analysis. | 9-10 |
| **Risk of bias across studies** | | 15 | | Specify any assessment of risk of bias that may affect the cumulative evidence (e.g., publication bias, selective reporting within studies). | 10 |
| **Additional analyses** | | 16 | | Describe methods of additional analyses (e.g., sensitivity or subgroup analyses, meta-regression), if done, indicating which were pre-specified. | 9-10 |
| **RESULTS** | | | | |  |
| Study selection | 17 | | Give numbers of studies screened, assessed for eligibility, and included in the review, with reasons for exclusions at each stage, ideally with a flow diagram. | | 10-11 |
| Study characteristics | 18 | | For each study, present characteristics for which data were extracted (e.g., study size, PICOS, follow-up period) and provide the citations. | | 10-11, Table1-3 |
| Risk of bias within studies | 19 | | Present data on risk of bias of each study and, if available, any outcome level assessment (see item 12). | | Table 4 |
| Results of individual studies | 20 | | For all outcomes considered (benefits or harms), present, for each study: (a) simple summary data for each intervention group (b) effect estimates and confidence intervals, ideally with a forest plot. | | 11-14 |
| Synthesis of results | 21 | | Present results of each meta-analysis done, including confidence intervals and measures of consistency. | | 11-14 |
| Risk of bias across studies | 22 | | Present results of any assessment of risk of bias across studies (see Item 15). | | 11 |
| Additional analysis | 23 | | Give results of additional analyses, if done (e.g., sensitivity or subgroup analyses, meta-regression [see Item 16]). | | 11-13 |
| **DISCUSSION** | | | | |  |
| Summary of evidence | 24 | | Summarize the main findings including the strength of evidence for each main outcome; consider their relevance to key groups (e.g., healthcare providers, users, and policy makers). | | 14-17 |
| Limitations | 25 | | Discuss limitations at study and outcome level (e.g., risk of bias), and at review-level (e.g., incomplete retrieval of identified research, reporting bias). | | 17 |
| Conclusions | 26 | | Provide a general interpretation of the results in the context of other evidence, and implications for future research. | | 18 |
| **FUNDING** | | | | |  |
| Funding | 27 | | Describe sources of funding for the systematic review and other support (e.g., supply of data); role of funders for the systematic review. | | 19 |

From: Moher D, Liberati A, Tetzlaff J, Altman DG, The PRISMA Group (2009). Preferred Reporting Items for Systematic Reviews and Meta-Analyses: The PRISMA Statement. PLoS Med 6(7): e1000097. doi:10.1371/journal.pmed1000097
