# Supplementary material for: Serum Aquaporin 4-Immunoglobulin G Titer and Neuromyelitis Optica Spectrum Disorder Activity and Severity: A Systematic Review and Meta-Analysis
Source: Front Neurol. 2021 Oct 20;12:746959. doi: 10.3389/fneur.2021.746959 (PMC8565925; doi:10.3389/fneur.2021.746959)
Supplement: Supplementary file 2 [file Data_Sheet_2.docx]

**Figure legends**

**Supplemental Figure 1.** The sensitive analysis of comparison of serum AQP4-IgG titer between the attack and remission phase in patients with NMOSD.
