# Supplementary figures and images for: Serum Aquaporin 4-Immunoglobulin G Titer and Neuromyelitis Optica Spectrum Disorder Activity and Severity: A Systematic Review and Meta-Analysis
Source: Front Neurol. 2021 Oct 20;12:746959. doi: 10.3389/fneur.2021.746959 (PMC8565925; doi:10.3389/fneur.2021.746959)

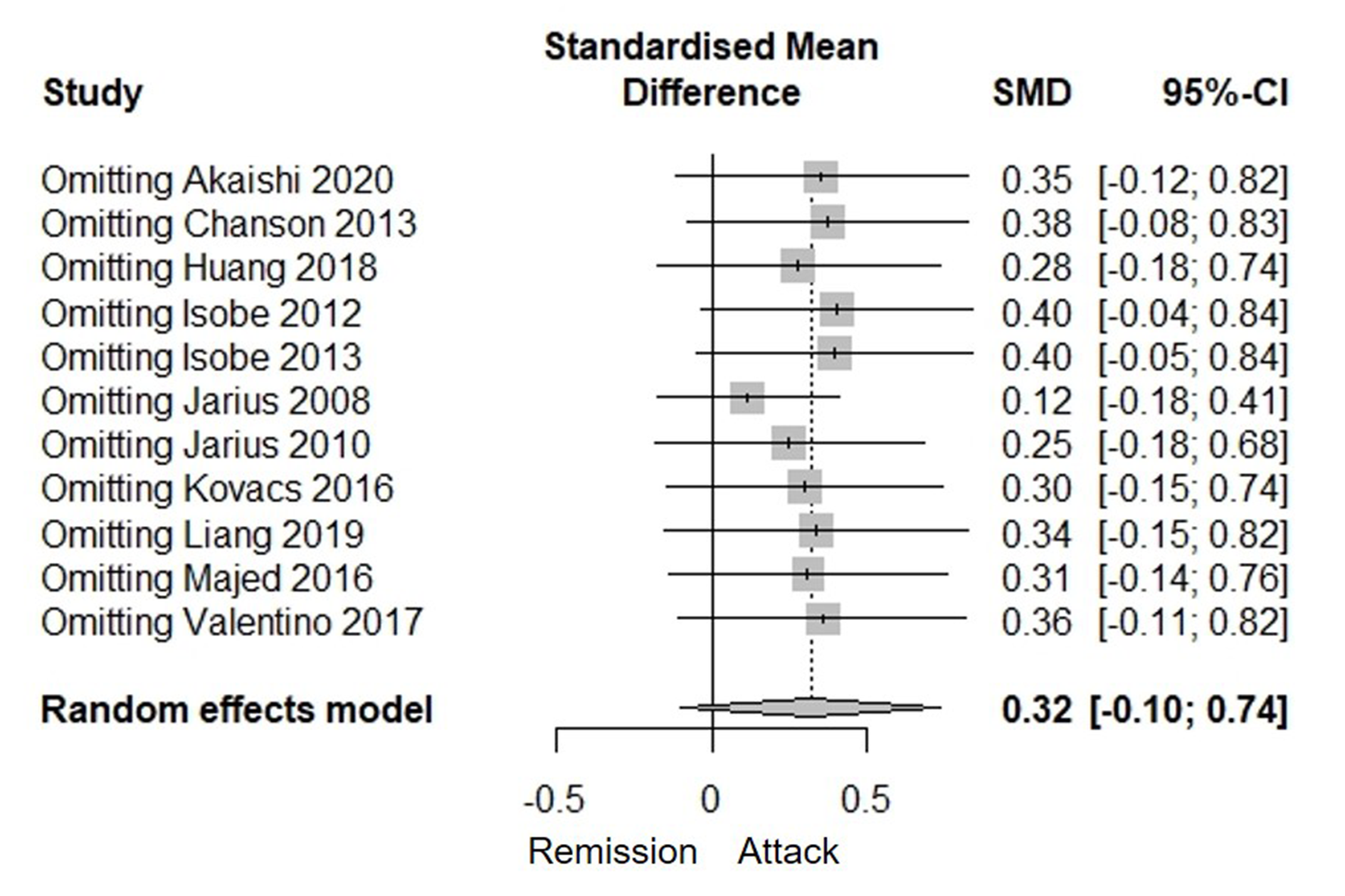

Supplement: Supplementary file 3 [file Image_1.TIF]
